# Evaluation of Safety and Efficacy of Salvage Therapy With Sunitinib, Docetaxel (Tyxane) and Cisplatinum Followed by Maintenance Vinorelbine for Unresectable/Metastatic Nonsmall Cell Lung Cancer: Stage 1 of a Simon 2 Stage Clinical Trial: Erratum

**DOI:** 10.1097/01.md.0000481355.09097.27

**Published:** 2016-03-03

In the article “Evaluation of Safety and Efficacy of Salvage Therapy With Sunitinib, Docetaxel (Tyxane) and Cisplatinum Followed by Maintenance Vinorelbine for Unresectable/Metastatic Nonsmall Cell Lung Cancer Stage 1 of a Simon 2 Stage Clinical Trial”,^1^ which appeared in Volume 94, Issue 52 of *Medicine*, the word tyxan was spelled incorrectly as tyxane. The article has since been corrected online.
